# An Investigative Study into Psychological and Fertility Sequelae of Gestational Trophoblastic Disease: The Impact on Patients’ Perceived Fertility, Anxiety and Depression

**DOI:** 10.1371/journal.pone.0128354

**Published:** 2015-06-01

**Authors:** Valentina E. Di Mattei, Letizia Carnelli, Martina Bernardi, Elena Pagani Bagliacca, Paola Zucchi, Luca Lavezzari, Veronica Giorgione, Alessandro Ambrosi, Giorgia Mangili, Massimo Candiani, Lucio Sarno

**Affiliations:** 1 Vita-Salute San Raffaele University, Faculty of Psychology, Milan, Italy; 2 Clinical and Health Psychology Unit, Department of Clinical Neurosciences, IRCCS San Raffaele Hospital, Milan, Italy; 3 Department of Obstetrics and Gynecology, IRCCS San Raffaele Hospital, Milan, Italy; Harvard Medical School, UNITED STATES

## Abstract

**Objectives:**

Gestational Trophoblastic Disease (GTD) comprises a group of disorders that derive from the placenta. Even if full recovery is generally expected, women diagnosed with GTD have to confront: the loss of a pregnancy, a potentially life-threatening diagnosis and delays in future pregnancies. The aim of the study is to evaluate the psychological impact of GTD, focusing on perceived fertility, depression and anxiety.

**Methods:**

37 patients treated for GTD at San Raffaele Hospital, Milan, took part in the study. The STAI-Y (*State-Trait Anxiety Inventory*), the BDI-SF (*Beck Depression Scale-Short Form*) and the FPI (*Fertility Problem Inventory*) were used. Patients were grouped on the basis of presence of children (with or without), age (< or ≥35) and type of diagnosis (Hydatidiform Mole, HM, or Gestational Trophoblastic Neoplasia, GTN). Differences in the values between variables were assessed by a t-type test statistic. Three-way ANOVAs were also carried out considering the same block factors.

**Results:**

The study highlights that women suffering from GTN had higher depression scores compared to women suffering from HM. A significant correlation was found between anxiety (state and trait) and depression. Younger women presented higher Global Stress scores on the FPI, especially tied to *Need for Parenthood* and *Relationship Concern* subscales. *Need for Parenthood* mean scores significantly varied between women with and without children too.

**Conclusions:**

We can conclude that fertility perception seems to be negatively affected by GTD diagnosis, particularly in younger women and in those without children. Patients should be followed by a multidisciplinary team so as to be supported in the disease’s psychological aspects too.

## Introduction

Gestational trophoblastic disease (GTD) comprises a group of disorders that derive from the placenta with varying tendencies for local invasion and metastases. They develop in the uterus after an abnormal pregnancy event. GTD includes all gestational trophoblastic diseases: Complete Mole, Partial Mole, Choriocarcinoma, Placental Site Trophoblastic Tumour (PSTT) and Epithelioid Trophoblastic Tumour (ETT) [1]. We shall divide these into Hydatidiform Mole (HM) and Gestational Trophoblastic Neoplasia (GTN). Hydatidiform Mole is an abnormal pregnancy composed of two distinct premalignant entities based on both morphologic and cytogenetic criteria: the Complete Mole and the Partial Mole. The malignant forms of GTD are also collectively known as Gestational Trophoblastic Neoplasia (GTN) which therefore include Choriocarcinoma, PSTT and ETT. GTN is treated with chemotherapy. GTN often arises after a molar pregnancy (around 0.5–1% of Partial Moles and 15–29% of Complete Moles may progress to GTN [2]) but can also occur after any gestational event, including term pregnancies. GTD was associated with significant mortality before the discovery of chemotherapy [3]; presently, GTNs are among the most curable of all solid tumours, with survival rates close to 100% [3].

Wide regional variations in the incidence of HM have been reported, ranging from 0.5–1 per 1000 pregnancies in North America, Australia and Europe to 1.5–6 per 1000 pregnancies in South America [3]. In Italy, the estimated frequency of HM over the 1996–2008 time period was 1 case every 935 pregnancies [4].

After suction and curettage for HM, a follow-up period is required to detect trophoblastic sequelae, such as GTN; patients are followed with weekly β human chorionic gonadotrophin (βhCG) levels until 3 consecutive normal values are obtained, then monthly for at least 6 months [5,3]. Chemotherapy is indicated when a plateau or a rise in βhCG occurs during the follow up period, or in presence of GTN. In this case, follow-up goes on for at least 1 year [2].

During βhCG follow up, patients are advised not to get pregnant (for at least 6 months after βhCG levels have normalised in a molar pregnancy, and for at least 12 months in any GTN that requires chemotherapy) [3]. This delay in future pregnancies, together with the experience of curettage and the GTD diagnosis, could elicit anxiety in many women, especially younger ones [5–7], and could negatively affect patients’ perceptions about fertility and the possibility of conceiving again in the future [7–9]. Wenzel and colleagues [8] observed that 40% of women treated successfully for GTD felt that they had no control on their reproductive future. Moreover, 17% felt angry that their ability to have children had been compromised. Another study [7] found that many patients were insecure (42%) or anxious (33%) before weekly βhCG controls; these patients scored higher for fear of recurrence, fear of infertility and fear of conceiving again, they were troubled by the advice to refrain from pregnancy during the follow-up period.

Using this literature as a starting point, we designed a study with the aim of evaluating how patients feel during the βhCG follow-up period after GTD. The chosen study measures were fertility-related concerns and the psychological symptomatology of depression and anxiety, which often characterise patients’ experiences during the monitoring period [5, 6,10,11]. We evaluated possible differences based on both clinical and socio-demographic characteristics: presence of children, age (< or ≥ 35) and type of diagnosis (Hydatidiform Mole or Gestational Trophoblastic Neoplasia).

Our selected study hypothesis posits that women who have a GTN rather than a HM diagnosis will have higher depression and anxiety scores and also higher fertility-related concerns [9]. Furthermore, we expect that women who already have children will have lower scores on all the study measures compared to nulliparous women [6]. Based on previous literature [12] we also hypothesise that younger women will have greater difficulty in adjusting to the illness and its consequences compared to older women.

## Materials and Methods

### Sample Selection and Recruitment

Patients treated for GTD at San Raffaele Hospital, Milan, were invited to take part in the research project. Eligible women had to be Italian-speaking, with at least an elementary school certificate and agreed voluntarily to participate in the research. Taking these criteria into consideration, 37 women took part in the study (n = 37). The study was approved by the Medical Ethical Committee of San Raffaele Hospital on May 6^th^, 2010. A written informed consent was obtained by all the participants at the time of questionnaire completion.

### Measures

Demographic and clinical information were collected by the use of a self-report questionnaire which included date of birth, level of education, relationship status, parity (current and prior to the GTD diagnosis), date of diagnosis, type of diagnosis and nature of therapy used to treat the disease.

Three validated questionnaires were also administered to assess psychological symptoms and fertility concerns.

The Beck Depression Inventory (BDI) [13] is one of the most widely used self-rating scales for measuring depression. Good reliability and validity of the BDI have been demonstrated in several studies [14]; in our research Cronbach’s alpha is in the range of 0.53–0.85 (α = 0.74). The BDI is composed of two subscales that measure cognitive-affective and somatic symptoms of depression, respectively [15]; the 13-item cognitive-affective subscale alone is known as BDI short form (BDI-SF) and is used to assess depression in the medically ill [16,17]. In the current study, the Italian version of BDI-SF was administered [18]. Responses are given on a 4-point Likert scale (from 0 to 3); a 9/10 cut-off score is suggested to detect moderate and severe depression in medical patients [17].

The Fertility Problem Inventory (FPI) [19] is a reliable and valid 46-item measure of perceived infertility-related stress. It provides information on five separate domains of patient’s concern (Table 1): social concern, sexual concern, relationship concern, need for parenthood and rejection of child-free lifestyle.

**Table 1:** FPI Subscales.

Social Concern	Feelings of discomfort and social isolation from one’s family and other people due to infertility-related problems; comparison with other people
Sexual concern	A perceived reduction of pleasure and self-esteem concerning one’s sexual life
Relationship concern	Difficulty in speaking about infertility with one’s partner and issues facing its impact on the relationship
Need for parenthood	Perception of parenthood as a fundamental target in life and a strong identification with the parental role
Rejection of child-free lifestyle	The perception that personal future satisfaction and happiness will rest on having a child

A measure of Global stress is also derived by summing the scores across all five subscales [20]. Responses are given on a Likert scale, from 0 (*strongly disagree*) to 6 (*strongly agree*); for women, mean scores greater than 27 indicate high levels of infertility-related stress [19]. All scales show good reliability, as indicated by the Cronbach's alpha reliability coefficient (social concern = 0.87; sexual concern = 0.77; relationship concern = 0.82; rejection of child-free lifestyle = 0.80; need for parenthood = 0.84; global stress = 0.93) [20]. In our study the Cronbach’s alpha reliability scores were as follows: social concern = 0.57 (0.49–0.65); sexual concern = 0.54 (0.29–0.70); relationship concern = 0.68 (0.50–0.81); rejection of child-free lifestyle = 0.66 (0.46–0.80); need for parenthood = 0.73 (0.56–0.84); global stress = 0.82 (0.70–0.89). The Italian version of FPI was used [21].

The State-Trait Anxiety Inventory (STAI) [22] is a widely used measure of state and trait anxiety. The STAI has good reliability (0.85 to 0.95), convergent and discriminant validity [23]. In the present research the state subscale Cronbach’s alpha was 0.89 (0.80–0.95) and the trait subscale was 0.83 (0.75–0.89). It has been used extensively in clinical contexts, including in the assessment of anxiety in cancer patients [24, 25]. It consists of two subscales each one composed of 20 items: the state subscale measures anxiety related to a specific situation or time-period (at the moment of questionnaire completion) while the trait one measures relatively stable anxiety (how one feels on a day-to-day basis). Responses are given on a 4-point Likert scale (from 1 to 4). Total scores range from 20 to 80 for each subscale; in order to provide clinical meaning, scores are grouped into three categories: low anxiety (scores of 20–39), medium anxiety (scores of 40–59) and high anxiety (scores of 60–80). In this study we used the Italian version of the STAI [26].

### Statistical analysis

The returned questionnaires were entered onto a spreadsheet database. Hereafter, the results were statistically analyzed using an R Statistical Environment (R Development Core Team, 2008) [27]. Categorical variables were summarized by means of percentages and numeric variables by mean value and standard deviation. Patients were grouped on the basis of presence of children (with or without), age (< or ≥ 35) and type of diagnosis (HM or GTN). Differences in the values between variables were assessed by a t-type test statistic. Correlations between numeric variables were evaluated by virtue of the Spearman’s correlation coefficient ρ_s_. Three-way ANOVAs were also carried out considering the same factors (age, diagnosis and presence or absence of children) to analyze the net effect of these factors on each scale. All p-values were computed by means of permutation methods [28] to avoid any distributional assumption and asymptotic approximation. The level of significance was set at p < .05.

## Results and Discussion

### Sample Characteristics

The age range of the sample was from 16 to 56 years (mean age = 35.6 years, S.D. = 10.4). Most of the women (70,3%; n = 26) were married, 16.2% (n = 6) were single, 8.1% (n = 3) were living with their partner and 5.4% (n = 2) were divorced. 59.5% (n = 22) of the women in our sample had had a child prior to the GTD diagnosis; whereas, for 40.5% (n = 15) this was their first pregnancy at the time of diagnosis. No patient declared to have had a child after GTD as all the women in our sample were in their βhCG follow-up period when the questionnaires were administered. With regards to the disease variables, most of the patients were suffering from Hydatidiform Mole (54%; n = 20), both partial and complete. The remaining women (46%; n = 17) were diagnosed with Gestational Trophoblastic Neoplasia (GTN), which included choriocarcinoma and placental site trophoblastic tumor (PSTT). The mean time elapsed from the moment of diagnosis to questionnaire completion was 6.6 months (range = 1–36; S.D. = 8.3). All patients with GTN were treated with chemotherapy, while women with HM diagnosis were undertaking only gonadotropin follow up.

### Anxiety and Depression

The mean score on the Beck Depression Scale was 5.4 (S.D. = 4.21), which does not indicate clinically significant depression; however, 6 of our 37 women (16.2%) showed levels of depression that can be considered severe (> = 9).

With respect to anxiety, there is a slight elevation in the state scale scores (mean = 42.7; S.D. = 10.1) compared to the trait scale ones (mean = 39.8; S.D. = 8.06). Both scores fall within the medium anxiety range of the STAI questionnaire (scores of 40–59), even if the trait scale mean is slightly below the 40 cut-off mark. Only 2 patients present high levels of state anxiety (scores ≥ 60), while no patient obtained high scores on the trait scale.

As shown in Table 2, significant differences in BDI-SF scores are found between women with HM and those with GTN (p = 0.03) and, consequently, between those who were undertaking chemotherapy and those who were not.

**Table 2:** BDI, STAI and FPI Global stress scores in different groups of patients (type of diagnosis; age; presence of children).

Type of diagnosis (Hydatidiform Mole, HM, or Gestational Trophoblastic Neoplasia, GTN)												
Group 0 = HM						Group 1 = GTN						
Variable	*n*.0	min.0	max.0	mean.0	SD.0	*n*.1	min.1	max.1	mean.1	SD.1	|t|	p-value
BDI total score	20	0	16	4.45	4.64	17	2	15	6.41	3.48	1.47	0.03*
STAI State score	20	25	65	41.6	9.43	17	30	72	44.4	11.0	0.84	0.48
STAI Trait score	20	25	56	38.8	8.00	17	29	52	41	8.23	0.84	0.44
FPI Global stress score	20	10	21	13.1	2.67	17	9	20	14.2	2.94	1.14	0.20
**Age (<or≥35)**												
Group 0 = patients<35 years						Group 1 = patients ≥ 35 years						
Variable	*n*.0	min.0	max.0	mean.0	SD.0	*n*.1	min.1	max.1	mean.1	SD.1	|t|	p-value
BDI total score	17	0	15	4.71	3.94	20	1	16	5.90	4.44	0.86	0.31
STAI State score	17	27	72	42.4	9.53	20	25	65	41.1	8.71	0.22	0.84
STAI Trait score	17	30	50	38.2	7.16	20	25	56	41.1	8.71	1.10	0.24
FPI Global stress score	17	10	20	14.7	2.46	20	9	21	12.7	2.79	2.40	0.01*
**Presence of children**												
Group 0 = without children						Group 1 = with children						
Variable	*n*.0	min.0	max.0	mean.0	SD.0	*n*.1	min.1	max.1	mean.1	SD.1	|t|	p-value
BDI total score	15	0	15	5.05	3.67	22	0	16	5.71	4.86	0.46	0.87
STAI State score	15	32	72	40.06	8.57	22	25	65	45.5	11.4	1.47	0.25
STAI Trait score	15	25	50	40.2	7.85	22	27	52	39.4	8.52	0.29	0.66
FPI Global stress score	15	10	20	13.7	2.56	22	9	21	13.5	3.16	0.25	0.77

*Statistical significant differences to the reference value (p<0.05)

On the contrary, anxiety and depression levels did not vary on the basis of time elapsed since diagnosis, presence of previous children and age (< 35 years). A significant correlation (Table 3) was also found between anxiety and depression: women with higher levels of anxiety (both state and trait) reported higher levels of depression (p<0.01 for both subscales).

**Table 3:** Correlations for the Study Population (N = 37).

		Spearman’s ρ_s_.	P-value
STAI state	BDI total score	0.53	< 0.01*
STAI trait	BDI total score	0.54	< 0.01*
FPI Global stress	STAI state	0.17	0.30
FPI Global stress	STAI trait	0.15	0.35
FPI Global stress	BDI total score	0.15	0.36
TIME passed since diagnosis	STAI state	-0.08	0.60
TIME passed since diagnosis	STAI trait	-0.08	0.62
TIME passed since diagnosis	BDI total score	0.01	0.92
TIME passed since diagnosis	FPI Global stress	0.14	0.40

*Statistical significant differences to the reference value (p<0.05)

### Infertility related stress

The Global stress mean score (mean = 13.6; S.D. = 2.81) is not of clinical significance (clinical significance is set between 27 and 30); the highest mean scores (range 0–6 for each subscale) were found in two different subscales of patients’ concern of the FPI, *Need for parenthood* (mean = 3.7; S.D. = 0.94) and *Rejection of child-free lifestyle* (mean = 3.5; S.D. = 0.96). A significant difference was found between younger (<35 years) and older women (≥35years) on the FPI Global stress scale (p = 0.01) (Table 2). To further investigate this aspect the various scales of the FPI questionnaire were separated out and analyzed (Table 4): *Need for parenthood* mean scores significantly varied on the basis of age (p<0.01).

**Table 4:** FPI subscale scores in patients < or ≥ 35 years.

Group 0 = patients <35 years						Group 1 = patients 35 ≥ years						
Variable	*n*.0	min.0	max.0	mean.0	SD.0	*n*.1	min.1	max.1	mean.1	SD.1	|t|	p-value
FPI Social concern scale	17	1.2	3.3	2.11	0.67	20	1	3.6	2.03	0.77	0.35	0.68
FPI Sexual concern scale	17	1	3.9	2.19	0.77	20	1	3.6	1.90	0.87	1.07	0.23
FPI Relationship concernscale	17	1.3	4.6	2.62	1.01	20	1	3.5	2.05	0.69	1.98	0.11
FPI Need for parenthood scale	17	2.9	5.8	4.16	0.77	20	1.6	5.2	3.30	0.90	3.16	<0.01*
FPI Rejection of child-free lifestyle scale	17	1.9	5.9	3.65	1.01	20	2	5.4	3.39	0.93	0.81	0.46

*Statistical significant differences to the reference value (p<0.05)

Three-way ANOVAs (Table 5) confirmed the effect of age on the Global Stress scale: younger women presented higher levels of Global Stress than older ones (p = 0.02).

**Table 5:** Three-way ANOVA of Global stress (FPI), Need for parenthood scale (FPI) Relationship concern scale (FPI).

Global Stress scale(FPI)			
	Estimate	F	p-value
Presence of children (> 0)	0.52	5.27E-06	0.99
Age (> = 35)	-2.17	5.91	0.02*
Diagnosis (GTN)	1.02	1.36	0.25
**Need for parenthood scale (FPI)**			
	Estimate	F	p-value
Presence of children (> 0)	-0.42	4.56	0.04*
Age (> = 35)	-0.77	7.30	0.01*
Diagnosis (GTN)	-0.05	0.03	0.84
**Relationship concern scale (FPI)**			
	Estimate	F	p-value
Presence of children (> 0)	0.33	0.42	0.51
Age (> = 35)	-0.64	5.46	0.02*
Diagnosis (GTN)	0.49	3.40	0.07

Block factors: presence of children, age (< or ≥ 35) years and type of diagnosis (Hydatidiform Mole, HM, or Gestational Trophoblastic Neoplasia, GTN)

*Statistical significant differences to the reference value (p<0.05)

Moreover, there is a significant effect of age on *Need for parenthood* and *Relationship concern* scales (p = 0.01 and p = 0.02 respectively, with younger women presenting higher scores). *Need for parenthood* mean scores significantly varied between women with or without children too (p = 0.04), with the latter showing higher scores.

Contrary to our expectations, there is no association between infertility-related stress, levels of anxiety and depression and time elapsed since diagnosis (Table 3).

### Discussion

Hydatidiform Mole and Gestational Trophoblastic Neoplasia are both highly curable diseases [2]. Despite the fact that full recovery is generally expected, women diagnosed with GTD have to confront the loss of a pregnancy, a potentially life-threatening diagnosis, surgical treatment and/or chemotherapy and delays in future pregnancies [29]. Even if the psychological impact of this condition for both the woman and her partner is clearly predictable and understandable, clinicians and health care professionals have often overlooked psychological distress in GTD and only recently more attention has been paid to the psychological effects of GTD [30].

All cultures throughout the ages have considered the ability to conceive and bear children as important to women. Historically, reproductive capacity has usually been closely tied to concepts of ‘femininity’ and gender identity; it has been stated that once a woman has been pregnant, there is no return to a to a “pre-pregnancy state of mind” [30]. The emotional impact of infertility in cancer has been widely studied in the past [31,32]. Most women report clinically significant levels of distress related to the loss of fertility, described as an emotionally devastating experience [32]. In GTD, even patients with widespread metastatic disease can expect to achieve remission while retaining their fertility[3,30]. However, the delay of future pregnancies due to βhCG levels follow-up could negatively affect patients’ perceptions about their possibility of conceiving again [7–9].

In our sample, even if levels of infertility-related stress (Global stress) were not of clinical significance, when women were divided by age group (with a cut-off of above or below 35 years), younger women presented higher scores than older ones. This result reflects other studies which show that younger women with cancer tend to report significantly greater concerns about infertility, premature menopause and menopausal symptoms [33–35], which enhance the level of distress and negatively affect adaptation to illness [36]. When the Global stress score was analysed and divided out into its different subscales, the effect of age is observed on the *Need for parenthood* scale too: younger women seem to be more concerned about the importance of parenthood in their lives; they may closely identify with the role of being a parent and parenthood seems to be perceived as an essential life goal (e.g. “*I will do just about anything to have a child*”). These infertility-related concerns have a role in the level of distress after treatment of GTD.

The three-way ANOVA not only highlights the effect of age, but also reveals an additional effect of the presence of children, which may have been hidden due to a confounding effect in the univariate analysis. Thus, we can conclude that women below 35 years and those who do not have children present higher scores on the *Need for parenthood* scale. These results aid in defining potential quality of life changes as a result of having had GTD: even though the GTD has been treated successfully, infertility-related concerns might persist, especially in younger women, and should be recognized as one of the most significant factors associated with the GTD experience [30]. Moreover, we may hypothesise that childless, younger women are driven by a stronger need to become a parent, thus the sudden interruption of their goal (due to the GTD diagnosis, treatment and follow-up) has a more negative impact on them. With regards to this aspect, Wenzel and colleagues [37] found that 47% of women suffering from GTD declared that after the treatment having a child was even more important, not only for themselves, but for their respective partners too.

The three-way ANOVA also highlights a significant effect of age on the *Relationship concern* scale, which analyses the presence of worries in communicating infertility issues with one’s partner and the impact that this may have on the couple. In our sample, younger women had higher scores on this scale than older ones: this result could suggest that these patients could have less consolidated relationships than older ones, thus finding it harder to share their worries and fears with their partners. Regarding this, Flam and colleagues [38] found that 71% of their GTD sample of women reported feeling abandoned by their partner, especially with regards to the management of the disease. 45% of women in their sample declared that the relationship with their partner had transformed into a “brother-sister” relationship. Furthermore, 5 couples in their sample of 22 had difficulties handling the stress of the illness and 4 couples separated. These results demonstrate the impact that GTD has on relationships. Despite a consistent pattern being found in the literature that most couples facing serious illnesses such as GTD find positive psychological changes in their relationships [9], this study shows how GTD could negatively impact the couple, especially when it comes to younger patients. Therefore we suggest including the partner as medical information and recommendations unfold after diagnosis [39].

The STAI questionnaire results showed that women in our sample experience significant levels of anxiety, both state and trait, which fall within the medium range. The higher scores in the state subscale could indicate that patients were particularly anxious about a situation that they perceived as dangerous at the time of questionnaire completion (they most probably attribute this to the GTD diagnosis, treatment and follow-up). Medium levels of anxiety are often present in cancer patient populations, especially situational anxiety tied to going to the hospital, waiting for medical appointments, waiting for diagnostic tests and results. [40]. With regards to GTD these findings are in line with previous studies that assert that fear of the disease, concerns about future pregnancies and waiting for the βhCG levels to normalize during follow-up can determine anxiety within these patients [6; 10; 11; 41]. On the contrary, discordant with other studies [6–9, 41] no differences in anxiety levels were observed between age groups (above and below 35 years of age).

With regards to depressive symptoms, although the overall BDI-SF mean score is below any clinical significance, 16.2% of our patients presented severe levels of depression. If we compare this percentage to the prevalence of depression in women with gynaecological cancer our results are in line with previous studies [42: 43] which found a prevalence of 23% of depression among this population of cancer patients.

In line with our study hypothesis, it is interesting to note that the univariate analysis revealed that women in the GTN diagnostic group had significantly higher depression scores (p = 0.03) compared to those with Hydatidiform Mole. As mentioned previously, patients with GTN undergo chemotherapeutic treatment, whereas those with HM do not. This result is consistent with previous cancer research whereby this difference may be due to more fatigue, more limitations in everyday activities and chemotherapy side effects, which could lead to greater pain and anguish in this group of women [44]. These women could therefore perceive their disease as more serious and perhaps feel a greater threat to their life. This is consistent with past GTD research, which indicates that women who require chemotherapy have greater mood disturbances and greater illness-related distress as well as have feelings of loss, anger, confusion and defectiveness [9,29]. However, after the three-way ANOVA there was no longer evidence of the effect of diagnosis on BDI scores. This may occur for two reasons. The first is that there was a confounding effect, whereby the outcome in the univariate analysis was biased by the unbalanced presence of another variable. The second reason is that it is possible that after conducting a three-way ANOVA some statistical power was lost given that the p-value in the univariate analysis was close to 0.05. This can be re-examined in the future with a larger sample size.

Correlations indicated that women with higher anxiety scores also presented higher depression scores. Anxiety and depression often correlate positively in cancer studies [45]. Moreover, in 2014 Wang and colleagues found that in breast cancer patients, anxiety and depression correlated. Higher levels of depression and anxiety were positively correlated with a higher level of passive coping style and negatively correlated with perceived social support, objective social support and an active coping style. It may be interesting in the future to conduct a similar study with GTD survivors.

In our study neither depression nor anxiety levels were influenced by the presence of children prior to the GTD diagnosis. This result contrasts our study hypothesis and previous research, which underlines this element as a protective factor for GTD patients, associated with significantly better psychological function and quality of life scores [6,11,37,39]. In this study we found that children are protective only in terms of infertility-related stress.

Several important limitations are acknowledged. It is noted that the sample is predominantly Caucasian, Italian and relatively well-educated and that women of different backgrounds may respond differently to these questionnaires. Furthermore, the study did not necessarily have a sufficiently large sample size (n = 37) to detect very subtle differences and this also makes it difficult to make generalizations on this small sample of women. However, due to the rarity of this disease it is difficult to recruit a large sample size. Furthermore, no registry was used to recruit patients but rather we preferred to administer the questionnaires individually to patients during their βhCG follow-up period. In the future, with a larger sample size it will be possible to divide women into the specific subtypes of GTD and not only into the two diagnostic groups used in this study.

The time elapsed since diagnosis varied greatly (from 1 to 36 months). This could be a potential problem since it is possible that women who were measured many months after their diagnosis and treatment adjusted differently to those newly diagnosed.

Moreover, this study was a cross-sectional evaluation of the psychological and infertility-related consequences of GTD. In the future, it may be possible to conduct a longitudinal study so as to follow the psychosocial effects of GTD from diagnosis to the end of follow-up and even beyond that, to develop predictive models to identify those most likely to benefit from additional psycho-educational efforts. Furthermore, it may be useful to develop a specific questionnaire for GTD-related infertility in Italian.

Notwithstanding these limitations, there are very few studies conducted in Italy to date on the psychological consequences of GTD and these descriptive data are important for enhancing our understanding of the patient experience of GTD, especially in a hospital setting.

These findings highlight the long-term psychosocial burden of a GTD diagnosis and should alert physicians to the importance of including a supportive care component in the clinical management of these women, even those who do not require chemotherapy. Particular subsets of patients that may require greater psychological input are younger women and those diagnosed with GTN that must undergo chemotherapy treatment. Considering that adolescents account for a substantial proportion of the population with GTD [46], clinicians must pay particular attention to educating younger women about their future fertility outcomes and options. The minimum standard of care should involve education related to the disease, to the treatment and its side effects, and reassurance related to generally favourable prognoses as well as ensuring that no deleterious effects will occur on subsequent pregnancies after a cure from GTD has been obtained. This would help to allay fears, enhance compliance and reduce quality of life and relationship disruption in these patients and their partners. We suggest a multidisciplinary approach be taken with all patients suffering from GTD.
